# Mn-Doped CaBi_4_Ti_4_O_15_/Pb(Zr,Ti)O_3_ Ultrasonic Transducers for Continuous Monitoring at Elevated Temperatures

**DOI:** 10.3390/s17122740

**Published:** 2017-11-27

**Authors:** Makiko Kobayashi, Taiga Kibe, Hajime Nagata

**Affiliations:** 1 Faculty of Advanced Science and Technology, Kumamoto University, Kumamoto 860-8555, Japan; daydreamtk@gmail.com; 2 Department of Electrical Engineering, Tokyo University of Science, Tokyo 162-0825, Japan; h-nagata@rs.noda.tus.ac.jp

**Keywords:** ultrasonic transducers, high temperature measurement, nondestructive testing, thin film sensor

## Abstract

Continuous ultrasonic in-situ monitoring for industrial applications is difficult owing to the high operating temperatures in industrial fields. It is expected that ultrasonic transducers consisting of a CaBi_4_Ti_4_O_15_(CBT)/Pb(Zr,Ti)O_3_(PZT) sol-gel composite could be one solution for ultrasonic nondestructive testing (NDT) above 500 °C because no couplant is required and CBT has a high Curie temperature. To verify the high temperature durability, CBT/PZT sol-gel composite films were fabricated on titanium substrates by spray coating, and the CBT/PZT samples were tested in a furnace at various temperatures. Reflected echoes with a high signal-to-noise ratio were observed up to 600 °C. A thermal cycle test was conducted from room temperature to 600 °C, and no significant deterioration was found after the second thermal cycle. To investigate the long-term high-temperature durability, a CBT/PZT ultrasonic transducer was tested in the furnace at 600 °C for 36 h. Ultrasonic responses were recorded every 3 h, and the sensitivity and signal-to-noise ratio were stable throughout the experiment.

## 1. Introduction

Ultrasonic monitoring at elevated temperatures is desired in industrial fields because it can detect failure phenomena such as thickness reduction, corrosion cracks, micro-void generation, and processed material deterioration in an early stage [1,2,3]. Even though ultrasonic nondestructive testing (NDT) is one of the common NDT techniques [4], long-term high-temperature application of ultrasonic NDT is difficult. For the general ultrasonic transducer using the contact method, an acoustic couplant to transmit ultrasound into test objects and a backing material to achieve a broad frequency bandwidth are used [5]. However, these materials cannot withstand elevated temperatures in the long term. Non-contact ultrasonic methods, such as air-coupled ultrasonic transducers [6], electromagnetic ultrasonic transducers (EMATs) [7], and laser-ultrasound [3], seem to be good solutions for these problems, but each technique has drawbacks. Air-coupled ultrasonic transducers have good acoustic impedance matching with air; therefore, it is suitable for the NDT of light materials such as graphite composites but not for the NDT of metals, which are the main targets of high-temperature ultrasonic NDT. EMATs have poor signal-to-noise ratios (SNRs) in general. The installation cost of a laser ultrasound system is high, and it is difficult to monitor many points of unmovable objects simultaneously. The use of a delay line is another method to avoid the direct contact of the ultrasonic measurement system with high temperatures [8], though the measurement system becomes bulky. Therefore, the development of an ultrasonic NDT method for high thermal resistance metals such as titanium, steel, and special alloys at high temperature is still desired.

Sol-gel composite ultrasonic transducers have been investigated for the industrial NDT applications [9,10,11,12,13,14,15,16]. Sol-gel composites consist of ferroelectric powders and a dielectric sol-gel solution. Sol-gel composite ultrasonic transducers are suitable for high temperature applications because they achieve a broad frequency bandwidth without any backing material owing to the presence of micro pores inside the sol-gel composite. If sol-gel composite films are directly fabricated on the test object, an acoustic couplant is no longer necessary since sol-gel composite films establish high acoustic bonding with the substrates during the fabrication process. Furthermore, it is possible to fabricate sol-gel composite films on targets with complex geometry by using the spray technique [10,11,12,13,14,15,16]. Therefore, sol-gel composite ultrasonic transducers are applicable for industrial ultrasonic NDT at elevated temperatures.

CaBi_4_Ti_4_O_15_(CBT)/Pb(Zr,Ti)O_3_(PZT) sol-gel composites, consisting of CBT powders and PZT sol-gel, have been developed for high-temperature applications above 500 °C [14]. CBT powders were chosen because of their high Curie temperature (~790 °C) [17]. A PZT sol-gel solution was selected because it simultaneously satisfies the requirements of a high dielectric constant and a relatively high Curie temperature. The authors found that Mn-doped CBT/PZT ultrasonic transducers showed a sufficient sensitivity and SNR at 550 °C for thickness measurements [14]. However, the maximum operating temperature has not been determined yet since Mn-doped CBT/PZT ultrasonic transducers operated successfully at the maximum operating temperature of the hotplate used in the previous experiment. Furthermore, the long-term durability was not tested. In this study, the maximum operating temperature and long-term high-temperature durability of CBT/PZT ultrasonic transducers were investigated [15,16].

## 2. Materials and Methods

**Figure 1:**
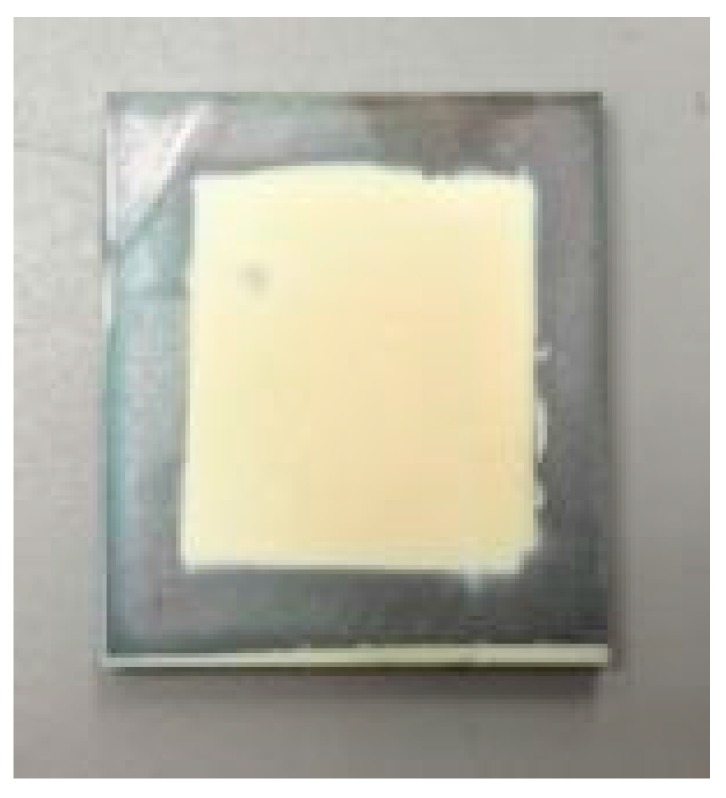
Optical image of 50-µm-thick and 400 mm^2^ Mn-doped CBT/PZT films fabricated on 3-mm-thick and 900 mm^2^ titanium substrate.

CBT/PZT films were fabricated on titanium substrates with a 3 ± 0.2 mm thickness, a 30 ± 1 mm length, and a 30 ± 1 mm width. Titanium was chosen as the substrate material because of its high-temperature durability and light weight so that its temperature quickly becomes equal to that of the furnace atmosphere. CBT/PZT films were manufactured via a sol-gel spray technique, and the details of the fabrication process can be found in elsewhere [9,10,11,12,13,14,15,16]. First, 0.5 wt % Mn-doped CBT raw material was manufactured by a conventional solid-state reaction method [14]. Then, synthesized CBT bulk ceramics was pulverized to fine CBT powders by a commercial milling device. CBT powders were mixed with a self-manufactured PZT sol-gel solution. The mixture was ball-milled for more than one day until a suitable viscosity for spray coating was achieved. When the mixture was ready for spray coating, the titanium substrates were covered with 80-µm-thick masks having windows 20 ± 1 mm in length and 20 ± 1 mm in width; then, the mixture of PZT sol-gel and CBT powders was sprayed on the titanium substrate with an air brush. After the spray-coating process, the thermal process, which involved drying at 150 °C for 5 min and firing at 650 °C for 5 min, was carried out. The spray-coating and thermal processes were repeated until the target thickness was achieved. In this study, these processes were repeated 5 times to obtain 50 ± 10-μm-thick CBT/PZT films. A typical optical image of the manufactured sample is shown in Figure 1.

A platinum top electrode with a diameter of 5 ± 0.5 mm was fabricated on each CBT/PZT sample. Platinum was used as the top electrode material because of its high temperature stability. Positive corona discharge was used for the poling process because a high electrical field could be applied on the porous piezoelectric films without dielectric breakdown. During the poling process, the samples were heated at 400 °C by a hotplate. Poling process of ferroelectric materials with high Curie temperatures is generally operated at elevated temperatures to supply electric field efficiently. In this experiment, poling process of CBT/PZT was carried out around 400 °C because the dielectric constant of the PZT sol-gel phase was nearly maximum around 400 °C of hot plate temperature and CBT ferroelectric phase could receive high enough electrical field. The piezoelectric constant d_33_ was measured using a commercial d_33_ meter, and it was determined to be 10 ± 1.5 pC/N for every sample in this study.

**Figure 2:**
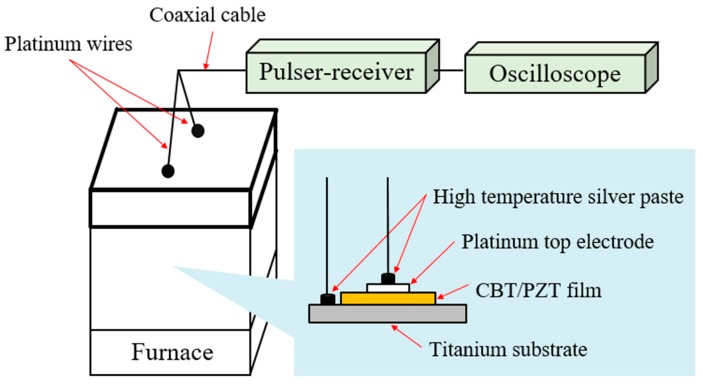
Schematic of ultrasonic measurement at elevated temperatures.

A schematic of the ultrasonic measurement is shown in Figure 2. Platinum wires were connected to both the platinum top electrode and the titanium substrate by a high-temperature silver paste. A CBT/PZT sample with platinum wires was placed in a furnace. Two platinum wires passed through the holes, which were created at the upper surface of the furnace, and were connected to the coaxial cable. The other side of the coaxial cable was connected to a pulser/receiver (P/R) machine to measure ultrasonic responses in pulse-echo mode. The output of the P/R machine was recorded using a digital oscilloscope.

## 3. Results and Discussions

### 3.1. Maximum Operating Temperature Test

**Figure 3:**
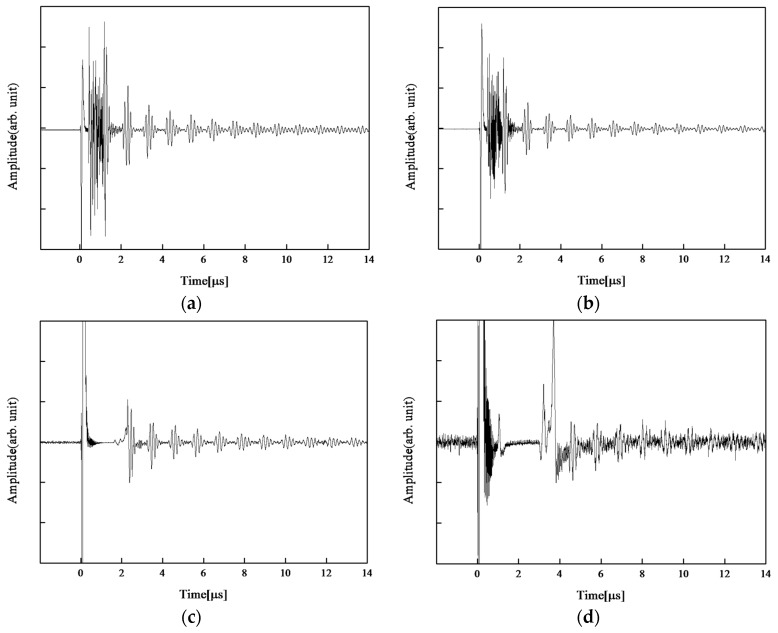
Ultrasonic measurement result of CBT/PZT films fabricated on 3-mm-thick 900 mm^2^ titanium substrate at (**a**) 200 °C, (**b**) 400 °C, (**c**) 600 °C, and (**d**) 700 °C.

The maximum operating temperature of Mn-doped CBT/PZT was determined. The center frequency and the 6 dB bandwidth of the fabricated ultrasonic transducer were 6.7 MHz and 4.5 MHz, respectively. Ultrasonic responses were recorded at room temperature (23 °C), 50 °C, and then for every 50 °C increase in temperature, up to 600 °C. Between 600 and 700 °C, the data were recorded for every 20 °C increase in temperature. A holding time of 5 min was set before data recording. This holding time was determined by a temperature measurement of a titanium substrate with neither film nor electrical connection by thermocouple prior to the experiment. Figure 3 shows the ultrasonic measurement results at 200 °C, 400 °C, 600 °C, and 700 °C, respectively. The dead-zone length varied intricately, and electrical impedance changed, presumably owing to the curing effect of the top electrode and resistance reduction of CBT at high temperatures. Clear reflected echoes were observed at all temperatures except 700 °C (Figure 3d). It is worth noting that SNR was significantly deteriorated in Figure 3d as well.

**Figure 4:**
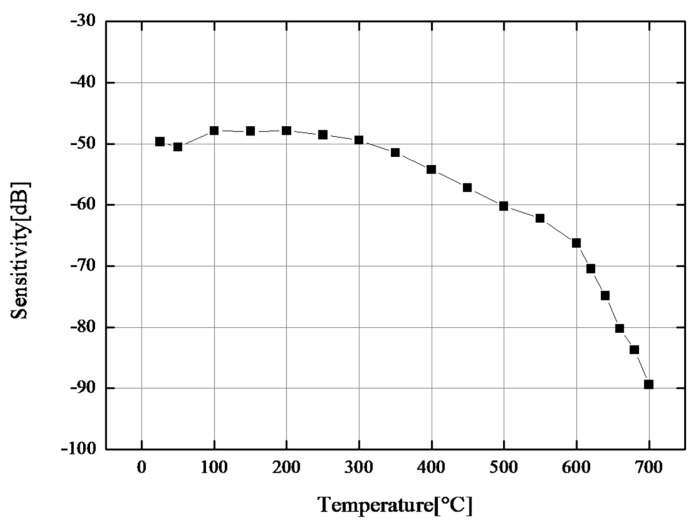
Sensitivity of CBT/PZT films fabricated on 3-mm-thick 900 mm^2^ titanium substrate at various temperatures.

In order to clarify the experimental results, the sensitivity *S* was calculated using the following equation:

(1) $$S=-\left\{\log_{10}(V_{1}/V_{2}+P/R\;Gain)\right\}$$

where *V*_1_ is the reference peak-to-peak voltage amplitude (V_p-p_), which was 0.4 V in this paper, and *V*_2_ is the amplitude of the third reflected echo. That is, the sensitivity *S* is determined as the negative value of P/R gain at which 0.4 V_p-p_ amplitude is achieved for the third echo. The fourth reflected echo was chosen because the first, second, and third reflected echoes disappeared at high temperatures because of the dead zone. The negative value was used to help intuitive understanding. The sensitivity at various temperatures is shown in Figure 4. At temperatures less than 300 °C, the sensitivity is very stable. The sensitivity *S* decreases linearly from 300 to approximately 600 °C, and it decreases exponentially beyond 600 °C. After cooling, the sensitivity *S* was much lower than the original value. From this result, we assumed that the breaking point of Mn-doped CBT/PZT ultrasonic transducers for thickness measurements is 600 °C. Based on this result, the maximum temperature was determined as 600 °C in the following experiments.

### 3.2. Thermal Cycle Test

**Figure 5:**
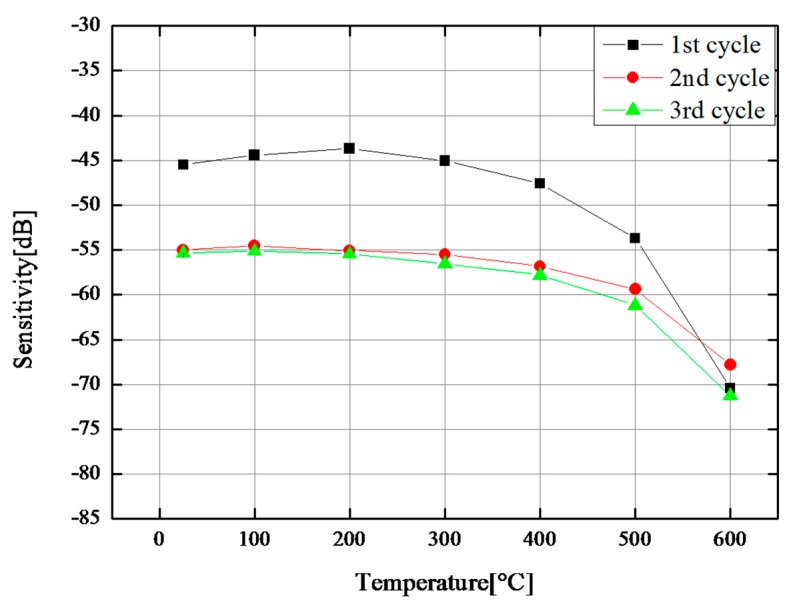
Sensitivity of CBT/PZT films fabricated on 3-mm-thick 900 mm^2^ titanium substrate at various temperatures during thermal cycle.

To ensure 600 °C durability, a thermal cycle test between room temperature and 600 °C was conducted for another CBT/PZT sample. The center frequency and 6 dB bandwidth of fabricated ultrasonic transducer were 6.8 MHz and 6.9 MHz, respectively. The measurement system was the same as that of the previous experiment. The ultrasonic responses were recorded in pulse-echo mode at room temperature, 100 °C, 200 °C, 300 °C, 400 °C, 500 °C, and 600 °C, after a holding time of 5 min for each temperature. During the cooling process, the furnace was turned off without ultrasonic monitoring. Figure 5 shows the sensitivity *S* at various temperatures during three thermal cycles. The sensitivity *S* was calculated using Equation (1) as in the previous section. The tendency was similar to that in Figure 4, i.e., the sensitivity *S* was stable at temperatures less than 300 °C, and beyond 300 °C the sensitivity decreased monotonically. The sensitivity *S* of CBT/PZT decreased after the first thermal cycle. We suggest that the irreversible depoling of the PZT sol-gel phase is the mechanism of sensitivity reduction after the first thermal cycle. The sensitivities *S* of the second and third thermal cycles were nearly identical. This reversible action mainly caused by the reversible depoling of the CBT powder phase and scattering loss of the titanium substrate. It should be mentioned that, after the third thermal cycle and following long-term ultrasonic monitoring, sensitivity *S* went back to its original value at room temperature. The SNR was stable between 15 and 20 dB. Therefore, CBR/PZT is suitable for thermal cycles ranging from room temperature to 600 °C.

### 3.3. Long-Term Ultrasonic Monitoring at 600 °C

**Figure 6:**
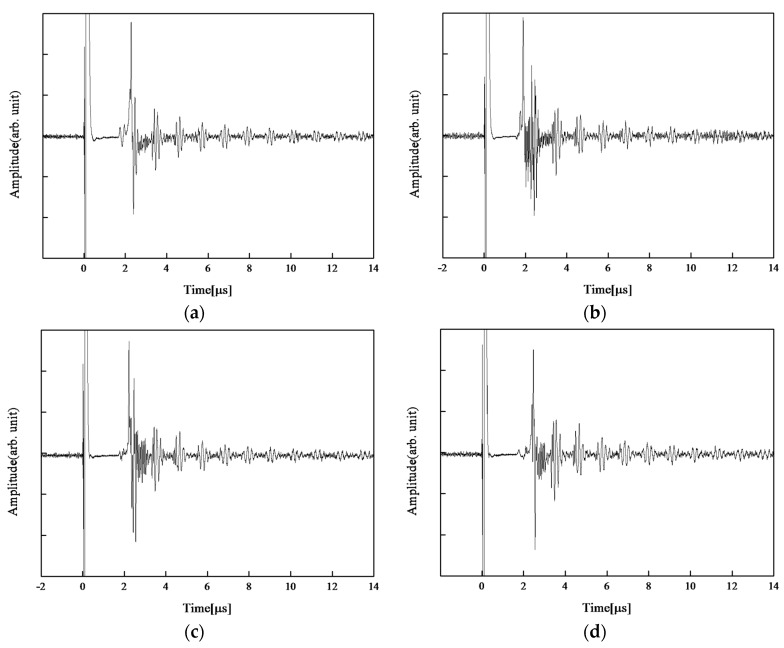
Ultrasonic measurement result of CBT/PZT films fabricated on 3-mm-thick 900 mm^2^ titanium substrate at 600 °C after (**a**) 0 h, (**b**) 6 h, (**c**) 24 h, and (**d**) 36 h.

**Figure 7:**
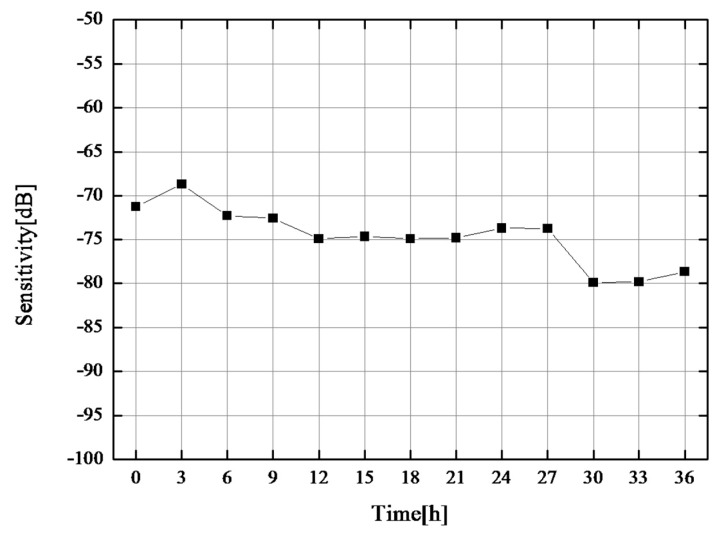
Sensitivity at 600 °C during the thermal durability test for 36 h.

After the third temperature rise, the furnace temperature was maintained at 600 °C for the long-term thermal durability test. Ultrasonic measurement in pulse-echo mode was performed every 3 h until 36 h passed. Figure 6 shows the ultrasonic responses of the CBT/PZT sample at 600 °C after 0 h, 6 h, 24 h, and 36 h, respectively. Even though the dead-zone length changed slightly, sufficiently clear multiple reflected echoes for thickness measurement were observed throughout the experiment. A change in dead-zone length would occur because of the deterioration of high-temperature silver paste, and further improvement is required for the electrical connection. It seems that the SNR was also stable at approximately 17 dB. Figure 7 shows the sensitivity at 600 °C during the thermal durability test for 36 h. The sensitivity was calculated using Equation (1) as in the previous section. The sensitivity was stable, and it could be concluded that CBT/PZT is a promising material for ultrasonic monitoring at 600 °C.

## 4. Conclusions

To confirm the ultrasonic measurement capability of CBT/PTZ at high temperatures, 50-µm-thick CBT/PZT sol-gel composite films with a ~400 mm^2^ area were fabricated on 3-mm-thick titanium substrates by the sol-gel spray technique. A platinum top electrode was fabricated on each film, and platinum wires were connected to both the platinum top electrode and the titanium substrate using a high-temperature silver paste. To determine the high-temperature durability of the manufactured transducers, one CBT/PZT sample was tested in the furnace from room temperature to 700 °C. It was found that CBT/PZT showed reasonable performance for thicknesses below 600 °C. The thermal cycle was repeated thrice between room temperature and 600 °C. The sensitivity lowered but became stable after the second thermal cycle. Subsequently, CBT/PZT was tested in the furnace at 600 °C for 36 h. The sensitivity of CBT/PZT was stable for 36 h. Therefore, it was concluded that the transducer made by the CBT/PZT sol-gel composite is promising for long-term monitoring in NDT applications at 600 °C.
